# News and Notes

**Published:** 1900-11

**Authors:** 


### *NEWS AND NOTES.*

Summer has gone with its languor and heat,
And soon will the wintry winds rankle—
Then Eberth's bacillus must take a back seat
For the pneumococcus of Fränkel.

THE *N. Y. Medical Journal* reports that of the 100 physicians practicing illegally in Missouri, about 40 of them are residents of Kansas City.

AT a meeting of the International Executive Committee of the Pan-American Medical Congress, Dr. William Perrin Nicolson was appointed secretary for the United States to the surgical section, a distinguished honor that is well merited. The congress will be held at Havana during the last week of the year.

TWO resolutions pertaining to the burial of the dead were recently introduced at a meeting of the Detroit Board of Health. The first provided that the dead from contagious diseases be cremated, as it is said it is a well-known fact that germs go into the soil and are a source of danger. The second resolution provided that the bodies of dead persons should be kept seventy-two hours before the undertaker is allowed to go to work, in order to prevent people being buried alive, or killed by embalming fluid. Both resolutions were tabled.

THE tenth annual conference of the American Electro-therapeutic Association was held recently at the Academy of Medicine, 17 West Forty-third street, this city. Papers relating to Electricity in Gynecology were read by Dr. Frederick D. Morse of Melrose,

Mass.; Dr. William J. Morton, Dr. F. Schavvir, of Stamford, Conn.; Dr. G. B. Massey of Philadelphia; Dr. Robert Newman of this city, and Dr. F. La Torre of Rome, Italy. The following officers were chosen for the current year: President, Dr. Ernest Wende of Buffalo; first vice-president, Dr. F. H. Morse of Melrose, Mass.; second vice-president, Dr. D. R. Brower of Chicago; secretary, Dr. G. G. Bill of Harrisburg; treasurer, Dr. R. G. Nunn of Savannah; chairman Executive Committee, Dr. Francis G. Bishop of Washington.

---

"DID you ever notice," said the barber, as I leaned back in his chair ready for a comfortable shave, while the electric fan by me blew a soft Atlantic City breeze, "what class of men always grow bald early in life, or suffer with thinness of the hair over the vertex of the head?" "No," said I, "the subject was never suggested to me before." "Well," said the barber, who was in a talkative mood, "I have been cutting hair for about twenty years and during this time I have made a study of the subject. My observation teaches me that baldness or thinness of the hair among young men is nearly always confined to those who live a very fast and dissipated life. To be more exact, it occurs most frequently among those men who indulge in sexual excesses, for among this class such indulgences seem to take away the very life of the hair, and it grows dry and brittle. For example," said he, "I have a friend whose hair for the past five years has been growing very thin just on top of his head. This young fellow had been going the 'gait that kills,' and had been living constantly with women. A year ago a member of his family died and since he has lived a very quiet, upright life. The result is, that when I cut his hair again on yesterday, I was utterly amazed at the new growth of hair and the new vitality displayed all over the scalp. He had done nothing whatever except to let his system recuperate a wasted strength."

"Will you have bay-rum or witch-hazel on your face?" said the barber, and I thought that even this class of men are philosophers.

R.

---

FOLLOWING is the preliminary program of the thirteenth annual meeting of the Southern Surgical and Gynecological Association, to be held in Atlanta, Ga., on November 13th, 14th and 15th, 1900:

1. President's Address—A. M. Cartledge, M.D., Louisville, Ky.

2. Trans-Peritoneal Drainage—L. S. McMurtry, M.D., Louisville, Ky.

3. A New Method Simplifying the Treatment of Complicated Fibroid Uteri and Severe Cases of Pelvic Inflammatory Diseases—Howard A. Kelly, M.D., Baltimore, Md.

4. A Report on a Case of Osteo-Fibro-Myoma of the Uterus—Geo. Ben Johnson, M.D., Richmond, Va.

5. The Open Treatment in Suppurating Forms of Appendicitis—Jos. Price, M.D., Philadelphia, Pa.

6. Aneurism of the Abdominal Aorta (Celiac Portion); Laparotomy; Isolation of Sac and wiring with twelve feet of silver wire; Electrolysis four and one-half hours. Death on nineteenth day, with remarks—Rudolph Matas, M.D., New Orleans, La.

7. A Flap Operation for Atresia of the Vagina, with Illustration—Geo. H. Noble, M.D., Atlanta, Ga.

8. Epi- and Hypospadias with Special Reference to their Operative Treatment—F. W. Parham, M.D., New Orleans, La.

9. Tuberculosis of the Female Pelvic Organs and their Treatment—Jos. Taber Johnston, M.D., Washington, D. C.

10. An Operation for Rectal Stricture—L. M. Tiffany, M.D., Baltimore, Md.

11. Value of Therapeutic Test in Two Cases of Syphiloma resembling Osteosarcoma—W. E. Parker, M.D., New Orleans, La.

12. Gastroenrostomy—F. T. Meriwether, M.D., Asheville, N. C.

13. Limitation in Abdominal Surgery—T. J. Crofford, M.D., Memphis, Tenn.

14. Early Excision for Dislocations not Reducible by Manipulation—W. F. Westmoreland, M.D., Atlanta, Ga.

15. A Study of Intra-Cranial Lesions—Hugh M. Taylor, M.D., Richmond, Va.

16. Carbolic Acid in Surgery—Seneca D. Powell, M.D., New York.

17. Drainage in Abdominal Surgery—J. W. Long, M.D., Salisbury, N. C.

18. Fracture of Shaft of Humerus Complicated with Dislocation—N. P. Dandridge, M.D., Cincinnati, O.

19. Etiology of Ovarian Dermoids—W. D. Haggard, Jr., M.D., Nashville, Tenn.

20. An Operation Devised for the Cure of Marked Rectal Prolapse in Women—J. Wesley Bovee, M.D., Washington, D. C.

21. Three Cases of Extra-Uterine Pregnancy—Walter B. Dorsett, M.D., St. Louis, Mo.

22. The Removal of Cystic Gall-Stones—Howard A. Kelly, M.D., Baltimore, Md.

23. Autointoxication from Defective Kidney Elimination, with and without Nephritis—Jas. T. Jelks, Hot Springs, Ark.

24. Pseudo-Membranous Enteritis, and its Relation to Surgery of the Abdomen—Frank A. Glasgow, M.D., St. Louis, Mo.

25. The Surgery of the Gasserian Ganglion—Wallace Neff, M.D., Washington, D. C.

26. Some Life-saving Measures in Obstetric Work, with Cases—R. R. Kime, M.D., Atlanta, Ga.

27. Anesthesia by Sub-Arachnoid Injection of Cocaine and Eucaine—W. L. Rodman, M.D., Philadelphia, Pa.

28. (a) Supplementary Report on Case of Litholapaxy. (b) Supplementary Report on Case of Vesico-rectal Fistula—Geo. S. Brown, M.D., Birmingham, Ala.

29. (* * * *)—A. J. Coley, M.D., Alexander City, Ala.

30. Appendicitis in the Female—Floyd W. McRae, M.D., Atlanta, Ga.

31. Cases of Ectopic Gestation—Jas. A. Goggans, M.D., Alexander City, Ala.

32. Traumatic Gangrene—J. B. Murfree, M.D., Murfreesboro, Tenn.

33. Solid Ovarian Tumors—John G. Earnest, M.D., Atlanta, Ga.

34. True Conservatism in the Management of Uterine Fibroids—Samuel E. Milliken, M.D., Dallas, Texas.

35. Report of a Case of Abdominal Pregnancy, with Specimen—R. B. Rhett, Jr., M.D., Charleston, S. C.

36. Surgical Treatment Immediately Following Labor, with Report of Cases—W. L. Robinson, M.D., Danville, Va.

37. Retro-Flexed Incarcerated Gravid Uterus—Wm. A. Quinn, M.D., Henderson, Ky.

38. Exsection of External Carotid Artery in Inoperable Disease of the Face, with Report of Cases—Wm. Perrin Nicolson, M.D., Atlanta, Ga.

39. Vesico-vaginal Fistulæ—M. C. McGannon, M.D., Nashville, Tenn.

40. Splenectomy, with Cases—W. E. B. Davis, M.D., Birmingham, Ala.

